# Re‐evaluating strategies for pollinator‐dependent crops: How useful is parthenocarpy?

**DOI:** 10.1111/1365-2664.12813

**Published:** 2016-11-11

**Authors:** Jessica L. Knapp, Lewis J. Bartlett, Juliet L. Osborne

**Affiliations:** ^1^ Environment and Sustainability Institute University of Exeter Penryn Campus Penryn Cornwall TR10 9FE UK; ^2^ Centre for Ecology and Conservation University of Exeter, Penryn Campus Penryn Cornwall TR10 9FE UK

**Keywords:** agricultural yield, agriculture, commercial crops, food security, fruit set, parthenocarpy, pollination, pollinator decline, pollinator dependence

## Abstract

1. Whilst most studies reviewing the reliance of global agriculture on insect pollination advocate increasing the ‘supply’ of pollinators (wild or managed) to improve crop yields, there has been little focus on altering a crop's ‘demand’ for pollinators.
2. Parthenocarpy (fruit set in the absence of fertilization) is a trait which can increase fruit quantity and quality from pollinator‐dependent crops by removing the need for pollination.
3. Here we present a meta‐analysis of studies examining the extent and effectiveness of parthenocarpy‐promoting techniques (genetic modification, hormone application and selective breeding) currently being used commercially, or experimentally, on pollinator‐dependent crops in different test environments (no pollination, hand pollination, open pollination).
4. All techniques significantly increased fruit quantity and quality in 18 pollinator‐dependent crop species (not including seed and nut crops as parthenocarpy causes seedlessness). The degree to which plants experienced pollen limitation in the different test environments could not be ascertained, so the absolute effect of parthenocarpy relative to optimal pollination could not be determined.
5. *Synthesis and applications*. Parthenocarpy has the potential to lower a crop's demand for pollinators, whilst extending current geographic and climatic ranges of production. Thus, growers may wish to use parthenocarpic crop plants, in combination with other environmentally considerate practices, to improve food security and their economic prospects.

## Abstract 2

Parthenocarpy has the potential to lower a crop's demand for pollinators, whilst extending current geographic and climatic ranges of production. Thus, growers may wish to use parthenocarpic crop plants, in combination with other environmentally considerate practices, to improve food security and their economic prospects.

## Introduction

Globally, agricultural land is continuing to expand and agricultural practices continue to intensify to meet rising food demands (Bommarco, Kleijn & Potts 2013). It is argued that sustainably maximizing agricultural yield requires ecosystem services to be optimized through improved soil quality, water efficiency and management of beneficial insects for pest control and pollination (Tilman *et al*. 2002; Bommarco, Kleijn & Potts 2013). Insect‐mediated pollination (the transfer of pollen within or between flowers via an insect) is a key regulating service for many crops and wild plants (Wilcock & Neiland 2002; Klein *et al*. 2007). Thus, any detrimental impact on pollination services, for example from habitat loss, introduced pests and diseases, and practices associated with intensive agriculture, could have a negative effect on crop yields and farmers’ profits (Steffan‐Dewenter *et al*. 2005; Potts *et al*. 2010; Goulson *et al*. 2015). Observed losses of pollinator species combined with our dependence on their contribution to food security have led to a widespread concern that we are facing a ‘pollinator crisis’ (Steffan‐Dewenter *et al*. 2002; Potts *et al*. 2010; although see Ghazoul 2005). However, whilst the plethora of recent reviews and studies on this subject come to similar conclusions that improving habitat and environmental conditions for pollinators will have a positive impact on crop production by increasing the ‘supply’ of pollinators (wild or managed), none of these studies consider the alternative option of reducing ‘demand’ for crop pollinators via technological innovation or management of crops. This can lead to a narrow (and potentially out‐dated) perspective given that, in the meantime, plant breeders and farmers are finding ways of short‐circuiting the need for pollination by developing and using new varieties which can set fruit without pollen vectors (Pandolfini, Molesini & Spena 2009).

The need for insect pollination in crops is usually measured in two ways: (i) pollinator dependence is quantified by comparing the yield of open‐ or hand‐pollinated crops with the yield of crops from which pollinators have been excluded. However, this is often only carried out for single cultivars in particular environmental conditions; (ii) pollination deficit estimates the additional pollination needed to achieve maximum yields in a particular context by comparing open‐pollinated with hand‐pollinated crops (Vaissière 2010). This technique has identified pollination deficits in a range of pollinator‐dependent crop species (See Table S1, Supporting Information) and is a vital step to evidence the need to implement management interventions to promote pollinator populations. Realistic estimates of the ‘value’ of insect pollination to global agriculture need to account for not only the variability in pollination deficit that might result from variable pollinator densities and environmental conditions, but also the variability in pollinator dependence between varieties of single crop species, for which there is currently little good evidence (Melathopoulos, Cutler & Tyedmers 2015). In the wider context, discussion and strategies for improving horticultural crop production (in particular) need to incorporate evidence on the variety of options available for increasing fruit and seed set by manipulating pollination systems, and not just assume that the only way to do this is by maximizing pollination. To improve estimates of pollinator dependence in crops and to widen the debate about how to guide farmers in improving seed and fruit production, we present a meta‐analysis of studies inducing parthenocarpy in horticultural crops.

Parthenocarpy (fruit set in the absence of fertilization) is a trait which has the potential to make many ‘pollinator‐dependent’ species produce fruit without pollination (Vardi, Levin & Carmi 2008). Parthenocarpy is thought to increase fruit quantity as plants are able to set fruit in conditions adverse for fertilization, for example due to poor pollen maturation or few pollinating species, typically seen in greenhouses or during periods of poor light and cold temperatures (Pandolfini 2009). Without parthenocarpy, and under these conditions, growers would ordinarily experience high rates of fruit abortion due to an insufficient number of pollen grains delivered to stigmas (Pandolfini 2009).

Parthenocarpy also has the potential to improve fruit quality as seedlessness (caused by no pollination and therefore fertilization) can be a desirable trait. This is different to stenospermocarpy, where seedlessness is achieved by seeds being aborted after fertilization (and therefore pollination) such as with triploid watermelons (Varoquaux *et al*. 2000). For example, it is thought to extend shelf life in some species, such as reduced browning in aubergine (Acciarri *et al*. 2002), is advantageous in fruit processing, such as tinned tomatoes (Pandolfini *et al*. 2002), and is generally favoured by consumers for convenience in preparation and consumption (Vardi, Levin & Carmi 2008). However, evidence suggests that some parthenocarpic plants may still produce a greater quantity and quality [including higher sugar content (Hayata *et al*. 2000; Shin, Park & Kim 2007)] of fruits when pollinated by insects (Robinson & Reiners 1999; Martínez *et al*. 2013; Nicodemo *et al*. 2013).

Fertilization of the ovules and seed/fruit development is co‐ordinated by various phytohormones, including auxins, gibberellins and cytokinins which originate from the developing embryos (Gillaspy, Ben‐David & Gruissem 1993). Phytohormones, present in developing seeds, are vital for regulating fruit growth and development (Gillaspy, Ben‐David & Gruissem 1993). However, in parthenocarpic (and therefore seedless) fruit set, endogenous phytohormones are elevated, suggesting that phytohormones from sources other than developing seeds can regulate fruit growth (Gustafson 1936). Consequently, parthenocarpy may be initiated through exogenous application of phytohormones. Auxins, gibberellins and cytokinins or mixtures of these have all been proven to be effective in inducing fruit development in the absence of fertilization and have been shown to increase productivity in various horticultural crops (Reviewed in Pandolfini 2009). However, little is known about the effect of these hormones on the environment and implementation is expensive and labour‐intensive (Saito *et al*. 2009). Consequently, scientists are increasingly finding ways to exploit genetic parthenocarpy.

Traditionally, approaches to genetic parthenocarpy have largely focused on selective breeding programmes for seedlessness (reviewed in Varoquaux *et al*. 2000; Vardi, Levin & Carmi 2008). For example, selective breeding of parthenocarpic sweet pepper (Tiwari, Dassen & Heuvelink 2007; Honda *et al*. 2012), papaya (Rimberia *et al*. 2007) and summer squash (Robinson & Reiners 1999; Kurtar 2003) varieties have all been shown to increase productivity. More recently, scientists have focused on genetic engineering approaches for parthenocarpic fruit set, through modification of auxin synthesis (iaaM), auxin sensitivity (rolB), auxin content (Aucsia), auxin signal transduction (iAA9 or ARF8) and gibberellin signal transduction (DELLA) (reviewed in Pandolfini 2009). For example, the chimeric auxin synthesizing DefH9‐iaaM gene has been shown to increase productivity in aubergine (Rotino *et al*. 1997; Donzella, Spena & Rotino 2000; Acciarri *et al*. 2002), tomato (Pandolfini *et al*. 2002; Molesini *et al*. 2009), cucumber (Yin *et al*. 2006), strawberry (Mezzetti *et al*. 2004) and raspberry (Mezzetti *et al*. 2004). Auxin‐synthesis parthenocarpy is facultative, meaning that it is seedless in conditions adverse for pollination/fertilization and seeded [although much reduced in number (Rotino *et al*. 2005)] in conditions where pollination occurs (Pandolfini, Molesini & Spena 2009). Breeding for genetic parthenocarpy also has the potential to combine multiple desirable traits. For example, parthenocarpy, female‐flowering time, improved fruit quality and disease resistance have been combined in cucumbers (Kushnereva 2008).

Using parthenocarpy to promote fruit set under unfavourable environmental conditions could improve the quality and quantity of pollinator‐dependent crops by reducing the number of poorly formed fruits caused by insufficient pollination (Pandolfini 2009). This could extend current geographic and climatic agricultural ranges of production, simultaneously improving food security and the economic prospects of commercial growers. Methods to induce parthenocarpy should therefore be taken into account when calculating the contribution of pollinators to fruit set, to avoid over‐estimating our dependence on them. Klein *et al*. (2007) provide the most comprehensive review of global crop pollinator dependence, and they acknowledge that their results are often based on studies from single cultivars and/or single regions because of the difficulty of finding comprehensive evidence. However, their data have been used to subsequently estimate the global value of pollination (Gallai *et al*. 2009; Breeze *et al*. 2011) and consequently justify the prediction of a ‘pollination crisis’ (Steffan‐Dewenter *et al*. 2005; Potts *et al*. 2010) without substantiated information at the individual crop level, as highlighted by Melathopoulos, Cutler & Tyedmers (2015).

In this study, we aim to go beyond previous reviews of parthenocarpy (Varoquaux *et al*. 2000; Gorguet, Van Heusden & Lindhout 2005; Vardi, Levin & Carmi 2008; Pandolfini 2009; Pandolfini, Molesini & Spena 2009) using meta‐analysis techniques to review and synthesize the literature on the extent of parthenocarpy‐promoting techniques currently being used commercially or experimentally on pollinator‐dependent crops across the world. Systematically reviewing plant science literature and applying it to pollination biology provides a broader perspective on the pollinator debate. We specifically investigate the following questions: (i) Does artificial or genetic parthenocarpy increase the quantity and quality of fruits in (normally) pollinator‐dependent crop species? (ii) Which method for conferring parthenocarpy: selective breeding, genetic modification or growth hormones, is most effective for parthenocarpic fruit set?

## Materials and methods

### Data Collection

We searched the ISI Web of Science, SCOPUS, Science Direct, Directory of Open Access Journals, AGRICOLA data bases and Google Scholar for studies that investigated the effect of genetic and artificial parthenocarpy on the quantity or quality of yield in pollinator‐dependent crops as defined by Klein *et al*. (2007), where pollinator dependence is classified as ‘essential’, ‘great’, ‘modest’ or ‘little’ (Table S1). Searches were conducted from 1945 to March 2016 using the search terms: (Parthenocarp*) AND (genetic mod* OR GM OR genetic* engineer* OR chimeric gene* OR selective breed* OR artificial selection OR hormone) AND (yield OR weight OR Brix). To avoid possible publication bias, patents were included and authors were emailed for relevant reports and unpublished studies (Koricheva, Gurevitch & Mengersen 2013).

Studies were included that met all the following criteria: (i) they were a pollinator‐dependent horticultural crop species; (ii) presented an effect of induced parthenocarpy on yield; (iii) reported the sample size; (iv) reported the mean, and if possible, the standard deviation for each treatment (for independent categorical variables). Methods to induce parthenocarpy were selective breeding or genetic modification (genetic parthenocarpy), or application of growth hormones (artificial parthenocarpy). Each intervention was compared to its own (negative) control. So, selective breeding compared parthenocarpic varieties with non‐parthenocarpic varieties (SB), growth hormones compared application with no application (HA), and genetic modification compared modified with non‐modified plants (GM). Effectiveness was measured in terms of crop quantity (e.g. weight per plant or yield) and quality in terms of sugar content (e.g. °Brix where one degree Brix is 1 g of sucrose in 100 g of nectar).

Authors of the original studies quantified the effect of parthenocarpy (i.e. compared parthenocarpic treatment with non‐parthenocarpic control) within different ‘test environments’ which can be broadly classified into hand pollination [this includes one example of experimental flowers being ‘selfed’, i.e. fertilized by pollen from the same plant (Molesini *et al*. 2009)] (hereafter, HP), no pollination (hereafter, NP) or open pollination (hereafter, OP). In both OP and HP conditions, only pollen from plants of the same genetic material were used. Conditions for which the plants were open pollinated vary between studies, from glasshouses supplemented with *Bombus terrestris* colonies to ‘open field’ conditions. The ecological complexity, or availability of pollinators at these ‘open fields’, was not provided. These test environments thus have differing background levels of potential pollination and were therefore included as a fixed effect in the analysis. The reasons for this were two fold: (i) to see whether NP conditions resulted in larger effect sizes (due to non‐parthenocarpic controls setting no fruit) and likewise smaller effect sizes in OP and HP conditions for the opposite reason (due to non‐parthenocarpic controls setting fruit) and (ii) to ensure that test environment did not influence treatment effectiveness. For OP and HP conditions to be included in the meta‐analysis, authors had to evidence parthenocarpic fruit set through either a much reduced number of seeds or that fruit set occurred in conditions adverse for pollinators (Pandolfini 2009).

### Calculation of Effect Sizes

Within individual studies different plant species, varieties and pure bred lines may be tested to determine which one has the best parthenocarpic potential for industrial development. Therefore, each genetic line was considered to be independent and thus included as separate cases in the data set. As a result, many studies contributed more than one entry to the data set. If a study examined multiple years or more than one treatment level of hormone concentration, then the largest sample size, or in cases with equal sample sizes the treatment level with the greatest effect, was selected.

Hedges’ *d* was used as a measure of effect size in our meta‐analysis. This measure is not affected by unequal sample sizes and includes a correction factor for small sample sizes (Koricheva, Gurevitch & Mengersen 2013). Hedges’ *d* was calculated for each treatment–control pair in the data set (see Table S2), based on the mean, standard deviation and sample size using the ‘metafor’ r package (Viechtbauer 2010).

We used bootstrapped analyses to fill in missing standard deviations (22 quantity samples and four quality samples), using 1000 resampled data sets following ‘hot deck’ imputation, outlined in Koricheva, Gurevitch & Mengersen (2013). We also include forest plots showing effect sizes using only complete data (without bootstrapping) in Figs S1–S3.

### Meta‐Analyses

Within a single attempt at inducing parthenocarpy, for example with multiple concentrations of hormones, the concentration which resulted in the greatest effect size (measured by hedges’ *d*) was selected. This was done to be representative of how these experimental studies would inform industry, where only the best lines and methods would be put forward for development.

All effect sizes were normalized for their positive skew using a real‐solution cube‐root transform (following Tukey's ladder of powers). To assess the importance of parthenocarpy‐inducing methods on crop quality and quantity, one sample two‐tailed t‐tests were used. The relative effectiveness of parthenocarpy‐inducing methods and the effect of different test environments were investigated with analysis of variance (anova). Possible interactions between these two effects were investigated with generalized linear models, using backward stepwise model selection (Crawley 2012).

## Results

Following a key word search of the literature, 161 studies investigated the effect of parthenocarpy in 33 pollinator‐dependent crop species. Of these, 35 did not supply full text, eight were not in English, and 78 used a study design unsuitable for inclusion (Table S1). The remaining data base included 184 effect sizes from 40 studies. Following our selection of the most effective treatments from each experiment (to reflect those which would be taken forward for development), our final sample size was 69 effect sizes (29 for genetic modification, 31 for hormone application and nine for selective breeding) (Table S2). These techniques had been used experimentally and/or commercially on 18 pollinator‐dependent crop species, of which three have an ‘essential’ need, six have a ‘great’ need, three have a ‘modest’ need, and three have a ‘little’ need for insect‐mediated pollination (three pollinator‐dependent species were unclassified) (Klein *et al*. 2007). Tomato was the most commonly studied species (16 studies), followed by aubergine (four studies) and sweet pepper (three studies). There was a notable absence of seed and nut crops; this was to be expected given that parthenocarpy causes seedlessness, an undesirable trait in these species. Likewise, an additional 14 pollinator‐dependent species showed no evidence of experimental or commercial parthenocarpy in the literature (Table S1).

**Figure 1:**
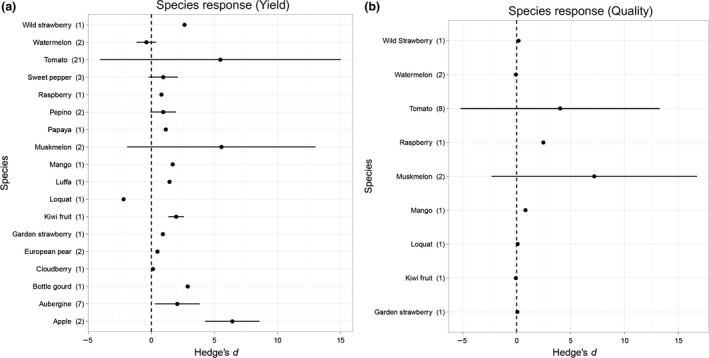
Mean effect sizes for all methods combined to induce parthenocarpy (genetic modification, hormone application and selective breeding) split by crop species (*y*‐axis) for (a) fruit quantity (b) fruit quality. Error bars represent standard deviations. Sample size (number of effect sizes) is given in parentheses.

All methods to induce parthenocarpy significantly increased fruit quantity (*t*_50_ = 8·41, *P* < 0·001) (Fig. 1a) and quality (*t*_17_ = 3·57, *P* = 0·002) (Fig. 1b). However, there were no significant differences in the effectiveness of genetic modification, selective breeding or hormone application for increasing fruit quantity (*F*_48_ = 0·41, *P* = 0·666) or quality (*F*_16_ = 0·86, *P* = 0·367). Test environment was shown to influence how effective treatments were on fruit quantity (*F*_48_ = 8·35, *P* < 0·001), with ‘no pollination’ environments having the largest effect size (Fig. 3). However, test environment did not influence the effectiveness of parthenocarpy‐inducing methods on fruit quality (*F*_15_ = 0·391, *P* = 0·683). Notably, there was no interactions between treatments and test environment (*F*_43_ = 1·63, *P* = 0·197), showing that the influence of test environments on treatment effectiveness was not biased against any particular parthenocarpy‐inducing method.

## Discussion

Artificial and genetic parthenocarpy have proven to be successful at increasing fruit quantity (Fig. 1a), without negatively affecting quality in all crop species studied (Fig. 1b). This is vitally important for commercial acceptance of parthenocarpy as it is only valuable to growers if there are no adverse effects on fruit quality, for example damaging normal vegetative growth (other than a reduced number of seeds) or a reduction in sugar and nutritional content (Pandolfini 2009). In this study, °Brix was used as a measure of quality as this was the only metric consistently recorded in studies.

**Figure 2:**
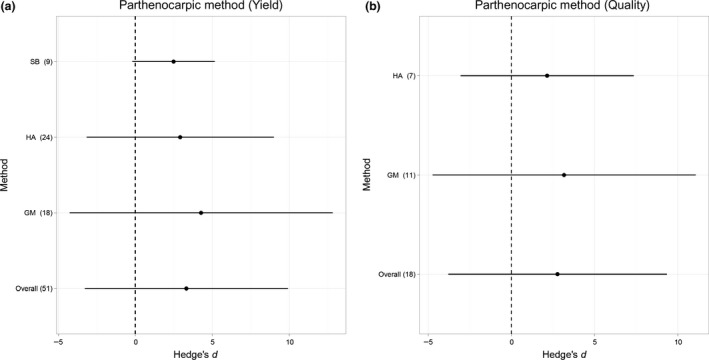
Overall mean effect sizes and effect sizes of methods to induce parthenocarpy [genetic modification (GM), hormone application (HA), selective breeding (SB)] (*y*‐axis) for (a) fruit quantity and (b) quality for all crop species. Error bars represent standard deviations. Sample size (number of effect sizes) is given in parentheses.

The most studied method for inducing parthenocarpy is hormone application, which was shown to positively increase crop quantity and quality (Fig. 2a,b). This method is currently the most widely used by commercial growers and although usually used prophylactically could be a very good temporary practice for periods of unfavourable environmental conditions.

Selective breeding (creating F1 hybrids) could provide a longer‐term solution for inducing parthenocarpy, which despite being investigated in fewer studies, still proved very successful at increasing yield (Fig. 2a,b). This complements yield trials not included in this meta‐analysis (see Table S1) which have found evidence of genetic parthenocarpy in pollinator‐dependent species. For example, 66% of sweet pepper varieties (Honda *et al*. 2012) and 33% of squash varieties examined (Robinson & Reiners 1999) were found to set parthenocarpic fruit. Although an effective method, selective breeding has its limitations. Principally, that crop species can only be crossed with ones that they can sexually reproduce with, and undesirable traits may be inherited alongside desirable ones during crossing. Likewise, selective breeding of varieties is expensive and time‐consuming, with varieties taking 5–10 years to be released (De Vries, Rabbinge & Groot 1997). This is because pure lines need to be maintained over many years to ensure their quality, and hybridization of pure lines often needs to be carried out by hand. Likewise, seeds grown from F1 hybrids often produce inferior yields to parental crops and consequently growers will need to purchase new F1 seeds each year (Tripp 1994).

Genetic modification for parthenocarpy could speed up this process by removing the need for back crossing and has been shown to be the most effective method in this meta‐analysis (Fig. 2a,b). This is supported by Donzella, Spena & Rotino (2000) who showed genetic modification to be more effective than hormone spraying at increasing yield. The authors concluded that genetic modification enabled a 10% reduction in production costs (less labour needed compared to hormonal sprays) and increased profit from improved quality following the genetic modification. Interestingly, genetic modification in strawberry and raspberry (Mezzetti *et al*. 2004), and tomato (García‐Hurtado *et al*. 2012; Medina *et al*. 2013) increased the number of flowers per plant, demonstrating the role that phytohormones also play in fecundity. Therefore, yield per plant may be greater than yield per fruit. Genetic methods could also use alternative methods of genetic engineering such as cisgenesis. This could increase the likelihood of regulatory and consumer acceptance by transferring genes between organisms that could otherwise be conventionally bred (Tester & Langridge 2010; Telem *et al*. 2013).

The range of effect sizes observed in this study (Fig. 2a,b) demonstrates the negative effects that unsuccessful parthenocarpy attempts can have on yield, alongside the highly positive effects that successful parthenocarpic treatments can have, for example those shown in tomato and muskmelon (Fig. 1a,b). The variation in the strength of these responses is primarily due to species‐specific responses to growth hormones (both applied and genetically modified). For example, if the expression of auxin coding transgenes (in genetically modified) or auxin concentration (from hormone application) is too high, then fruit may appear malformed, particularly in auxin sensitive species (Gorguet, Van Heusden & Lindhout 2005; Gemici, Türky?lmaz & Tan 2006). Likewise, relationships between different phytohormones are complex and vary greatly depending on species. This demonstrates the need for continued, multitreatment experiments to test the most effective strengths and types of hormones, tailored to individual crop species.

**Figure 3:**
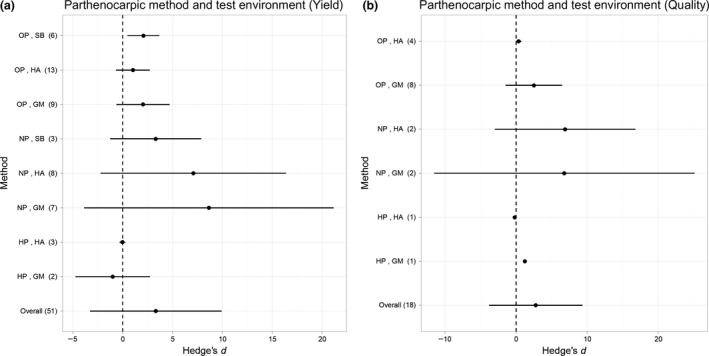
Overall mean effect sizes and effect sizes of methods to induce parthenocarpy [genetic modification (GM), hormone application (HA), selective breeding (SB)] and test environment (NP, OP and HP) (*y*‐axis) for (a) fruit quantity (b) fruit quality for all crop species. Error bars represent standard deviations. Sample size (number of effect sizes) is given in parentheses.

Investigating fruit quality and quantity in different test environments can allow us to assess how useful parthenocarpy could be in the total absence of pollination and fertilization. In the example of genetically modified aubergine, Acciarri *et al*. (2002) found a 30–35% increase in productivity, without any effect on quality under both greenhouse and open field conditions. In both test environments, the fruit was always seedless, therefore, positively influencing fruit quality and the economic value of production. Larger effect sizes in no pollination conditions (Fig. 3a,b) demonstrate the greater effect that parthenocarpy will have in conditions where fruit set would ordinarily be very low. Consequently in conditions where hand pollination is required for improved fruit set, artificial and genetic parthenocarpy could be a cost‐effective alternative (Allsopp, de Lange & Veldtman 2008; Niu, Wang & Li 2015). Conversely, effect sizes tend to be smaller in open‐ and hand‐pollinated environments where pollen is available (Fig. 3a,b). This is likely to be because in these conditions, the non‐parthenocarpic controls are successfully pollinated to some extent. However, in all test environments, plants may have experienced some pollination deficit (i.e. if plants were selfed, pollinated from just one donor plant, or if experiments were conducted in areas with low pollinator abundance). It is not possible to ascertain the degree of pollination deficit in the HP and OP test environments, and to what extent these limitations represent real‐world growing conditions. So, these results may over‐estimate the effect of parthenocarpy compared to yield resulting from open pollination in an environment where pollinators are not limiting, and natural pollination is thus optimal.

Nonetheless, parthenocarpy could still be useful in open pollination environments, where it can minimize the potential for pollination deficits whilst improving fruit uniformity caused by stochastic poor pollination (Pandolfini 2009). Therefore, parthenocarpy could be advantageous to all crops, whether or not they are experiencing a pollination deficit. In return, these parthenocarpic crops can continue to provide valuable nectar and pollen resources for our wild and managed bees, and other flower‐visiting insects. However, there is no information available as to how the quality and quantity of nectar and pollen varies between parthenocarpic and non‐parthenocarpic plants, or how selective breeding for parthenocarpy will affect a plant's nectar and pollen production over time. It is also worth remembering that parthenocarpic fruit set, and therefore seedlessness, is not always desirable, such as crop species where seeds are the edible part and for creating of seed stock.

Incomplete routes of communication between the plant breeding industry, ecologists and apiculturists have resulted in a mixed and potentially inaccurate message about the extent of our dependence on pollinators for food production (Ghazoul 2005; Kleijn *et al*. 2015; Melathopoulos, Cutler & Tyedmers 2015). Studies which value the contribution of insects to pollination are based on pollinator dependence, that is the extent that a plant depends on pollinators for fruit set. However, this metric assumes that dependence is constant within a single crop (Klein *et al*. 2007; Gallai *et al*. 2009). In reality, pollinator dependence is strongly dependent on variety, the spatial and temporal context of the surrounding landscape, and the responses of farmers, consumers and technological innovation to pollinator decline. Therefore, we highlight that there may be over‐estimation of pollinator dependence if studies overlook research and development currently underway to reduce the need for pollination. We found evidence for studies inducing parthenocarpy in four of 13 of the global crops for which pollination is considered essential (according to Klein *et al*. 2007); and 13 of 30 of the crops for which the need for pollination is considered great. This indicates that research into reducing *demand* for pollination has occurred in 40% of the crops for which ecologists are currently primarily only advocating an increase in *supply* of pollinators as the solution to improving crop yields and quality (Garibaldi *et al*. 2011; Carvalheiro *et al*. 2013). Indeed, there are three crop species in the top twenty crops for global production (Mt year^−1^ in Klein *et al*. 2007) which benefit from insect pollination and appear in this meta‐analysis of parthenocarpy studies (tomato #12; watermelon #15; apple #19). It is not surprising that, if a crop is showing a yield deficit, then different routes are explored to solve the problem, but it is surprising that evidence of the effectiveness of different approaches is not brought together more comprehensively to build an accurate picture for a crop. Single successes presented in this meta‐analysis could lead to profound changes in production of certain crops; for example, nearly all bananas on the global market are of the Cavendish variety, selectively bred to be parthenocarpic.

Data are not currently available to assess variety choice by farmers, or the level of parthenocarpy in the varieties that they choose. The results of this meta‐analysis support the conclusions of Klein *et al*. (2007) and Melathopoulos, Cutler & Tyedmers (2015) that to get a more complete picture, varietal information is required – both in terms of pollinator dependence, but also in terms of choices that farmers are making.

### Synthesis and Applications

Parthenocarpy may be able to reduce the need for pollinators in many horticultural crops but should not be used as a panacea for agricultural success. Biodiversity decline in agricultural landscapes is often an indicator of poor ecosystem health, which can also cause poor fruit set. Thus, agricultural growers should carefully consider causes of poor fruit set and ideally use parthenocarpic species (which can still provide an important nectar and pollen source for pollinator species) in addition to other environmentally considerate practices. Likewise, parthenocarpy could further the pollinator crisis by removing the imperative for conserving pollinators as our ‘dependence’ on them is reduced (Brown *et al*. 2016). This could affect pollination of non‐parthenocarpic pollinator‐dependent crops as well as wild plants. Ultimately, widespread implementation of these practices will be limited to countries that have access to, and can afford skilled personnel and equipment. Thus, free communication of resources and capabilities from developers to users is essential for the benefits of parthenocarpy to reach the areas of the world that are most in need of its benefits.

This study shows that genetic and artificial parthenocarpy has a great potential to improve fruit quantity, without affecting quality in a range of horticultural crops. Potentially, the most promising method for inducing parthenocarpy is genetic modification; the most effective for increasing fruit quality and quantity, whilst being the quickest to implement. However, whilst acceptance for genetic modification, particularly in Europe, remains equivocal, selective breeding may be a more attainable way for achieving genetic parthenocarpy. This method is also relatively cost‐effective for many horticultural growers already growing hybrid varieties. Although currently a popular choice, hormone application remains an expensive and un‐sustainable option for many horticultural growers. Nonetheless, any additional costs for agricultural growers associated with implementing genetic and artificial parthenocarpy could be offset by increasing the quality and quantity of crops. Unfortunately, no studies have directly compared the cost of parthenocarpy to traditional methods of supplemented pollination, such as introduced honeybee hives and hand pollination. Climate change could also increase pressure to develop parthenocarpic crop species as changes in pollinator distributions or declines in their populations are likely to be detrimental to food production (Kerr *et al*. 2015). Thus, parthenocarpic crop plants could allow producers to extend their growing seasons in otherwise adverse climatic and environmental conditions, furthering their economic advantage, increasing agricultural resilience and improving food security.

## Authors' contributions

J.K. and J.O. initiated the idea. J.K. designed the study, prepared the data and wrote the first draft of the manuscript. L.B. checked and analysed the data. J.K., L.B. and J.O. all contributed substantially to revising the manuscript.

## Data accessibility

The data supporting the results are in Table S2 of the Supporting Information and have been gathered from the associated references.

## Supporting information


**Fig. S1.** Mean effect sizes for all methods combined to induce parthenocarpy (genetic modification, hormone application, and selective breeding) split by crop species (y axis) for (a) fruit quantity (b) fruit quality.
**Fig. S2.** Overall mean effect sizes and effect sizes of methods to induce parthenocarpy [genetic modification (GM), hormone application (HA), selective breeding (SB)] (y axis) for (a) fruit quantity and (b) quality for all crop species.
**Fig. S3.** Overall mean effect sizes and effect sizes of methods to induce parthenocarpy [genetic modification (GM), hormone application (HA), selective breeding (SB)] and test environment (NP, OP, and HP) (y axis) for (a) fruit quantity (b) fruit quality for all crop species.

**Table S1.** Pollinator dependent crops, as defined by Klein *et al*. (2007) and studies (identified by the key word search) which investigate methods to induce parthenocarpy.

**Table S2.** Final dataset used in the meta‐analysis, alphabetically ordered by genus.
