# Supplementary material for: Re‐evaluating strategies for pollinator‐dependent crops: How useful is parthenocarpy?
Source: J Appl Ecol. 2016 Nov 11;54(4):1171–9. doi: 10.1111/1365-2664.12813 (PMC5516152; doi:10.1111/1365-2664.12813)
Supplement: Supplementary file 1 — Fig. S1. Mean effect sizes for all methods combined to induce parthenocarpy (genetic modification, hormone application, and selective breeding) split by crop species (y axis) for (a) fruit quantity (b) fruit quality. Fig. S2. Overall mean effect sizes and effect sizes of methods to induce parthenocarpy [genetic modification (GM), hormone application (HA), selective breeding (SB)] (y axis) for (a) fruit quantity and (b) quality for all crop species. Fig. S3. Overall mean effect sizes and effect sizes of methods to induce parthenocarpy [genetic modification (GM), hormone application (HA), selective breeding (SB)] and test environment (NP, OP, and HP) (y axis) for (a) fruit quantity (b) fruit quality for all crop species. [file JPE-54-1171-s001.docx]

**Figures S1-S3** Forest plots showing effect sizes only from studies with complete data, i.e. without bootstrapping for missing standard deviations

a)

b)

**Figure S1** Mean effect sizes for all methods combined to induce parthenocarpy (genetic modification, hormone application, and selective breeding) split by crop species (y axis) for (a) fruit quantity (b) fruit quality. Error bars represent standard deviations. Sample size (number of effect sizes) are given in parentheses.

a)

b)

**Figure S2** Overall mean effect sizes and effect sizes of methods to induce parthenocarpy (genetic modification (GM), hormone application (HA), selective breeding (SB)) (y axis) for (a) fruit quantity and (b) quality for all crop species. Error bars represent standard deviations. Sample size (number of effect sizes) are given in parentheses.

a)

b)

**Figure S3** Overall mean effect sizes and effect sizes of methods to induce parthenocarpy (genetic modification (GM), hormone application (HA), selective breeding (SB)) and test environment (NP, OP, and HP) (y axis) for (a) fruit quantity (b) fruit quality for all crop species. Error bars represent standard deviations. Sample size (number of effect sizes) are given in parentheses.
