# Supplementary material for: Re‐evaluating strategies for pollinator‐dependent crops: How useful is parthenocarpy?
Source: J Appl Ecol. 2016 Nov 11;54(4):1171–9. doi: 10.1111/1365-2664.12813 (PMC5516152; doi:10.1111/1365-2664.12813)
Supplement: Supplementary file 2 — Table S1. Pollinator dependent crops, as defined by Klein et al. (2007) and studies (identified by the key word search) which investigate methods to induce parthenocarpy. [file JPE-54-1171-s002.docx]

## **Table S1** Pollinator dependent crops, as defined by Klein et al. (2007) and studies (identified by the key word search) which investigate methods to induce parthenocarpy.

Crop species with no references demonstrate current areas where parthenocarpy has not been used commercially or experimentally. References in bold are included in the meta-analysis, references in black were identified in the systematic review but not included in the meta-analysis (i.e. they did not fulfil all of the search requirements), and references in blue only had an abstract available.

Species were pollinator dependent if animal pollination was shown to increase production (fruit set, fruit weight and/or quality, seed number and/or seed quality, and/or increased pollen deposition (an indirect measure)) for at least one variety per crop (Klein *et al.* 2007). Only crop species with essential, great, modest, and/or little dependence on pollinators were included in analyses. Essential = pollinators essential for most varieties (production reduction by ≥ 90% comparing experiments with and without animal pollinators. Great = great production increase/ animal pollinators are strongly needed (40 - <90 % reduction). Modest = modest production increase/ animal pollinators are clearly beneficial (10 -<40 % reduction) (Klein *et al.* 2007).

Seed and nut crops are absent from this table as parthenocarpy (therefore, seedlessness) would not be a desired trait. Entries are alphabetically ordered by genus.

|  |  | From Klein et al 2007 | | Studies which promote the effect of parthenocarpy on yield identified found from the systematic review of the literature. Studies in bold were included in the meta-analysis. | | |
| --- | --- | --- | --- | --- | --- | --- |
| **Crop species** | **Crop name** | **Requirement for animal pollination** | **Rank# in list of top global crops Mt/yr** | **Selective breeding** | **Genetic engineering** | **Growth hormones** |
| *Abelmoschus esculentus* | Okra | modest | #56 |  |  |  |
| *Actinidia deliciosa* | Kiwifruit | essential |  |  |  | **Iwahori *et al.* 1988**  Ohara et al. 1997 |
| *Annona squamosa* | Atemoya, Cherimoya, Custard apple | essential |  |  | Lora *et al.* 2011 |  |
| *Asimina triloba* | Pawpaw, Indiana banana | essential |  |  |  |  |
| *Averrhoa carambola* | Carambola, Starfruit | great |  |  |  |  |
| *Capsicum annum, C. frutescens* | Chilli pepper, Red pepper, Bell pepper, Green pepper, Allspice, Pimento | little | #33 | Carrizo 2011  Honda *et al.* 2012  **Tiwari *et al.* 2007** |  | Balakbir *et al.* 1998 Gustafson 1936 Heuvelink & Korner 2001  Thanopoulos *et al.* 2013  **Tiwari *et al.* 2012** **Wien & Zhang 1991** |
| *Carica papaya* | Papaya | little | **#52** | **Rimberia *et al.* 2007** |  |  |
| *Citrullus lanatus* | Watermelon | essential | #15 |  |  | **Hayata *et al.* 1995**  Huitrón *et al.* 2007  Kwon *et al.* 2006  **Maroto *et al.* 2005**  Sedgley *et al.* 1977  Hayata et al. 1994  Hikosaka et al. 2015  Newbury et al. 1977  Pak 1993  Miguel et al.2000 |
| *Citrus* spp.  *parthenocarpy listed as one of breeding mechanisms | Bergamont, Chinotto, Citron, Clementine, Grapefruit, Kumquat, Lemmon, Lime, Manderine, Orange, Pomelo, Tangerine | little | #13 | Mesejo *et al.* 2013 |  | García-Martínez & García-Papí 1979  Guardiola *et al.* 1993  Talon et al. 1992 |
| *Cucumis melo* | Cantaloupe, Melon | essential | #29 |  |  | **Hayata *et al.* 2000** Risser 1976  Masuda 1990 |
| *Cucumis sativus* | Cucumber, Gherkin | great | #24 | Kushnereva 2008  Li *et al.* 2014  Shaw *et al.* 2007  Sun *et al.* 2006  Yan *et al.* 2012  Dean et al. 1983  Dennijs et al. 1991 | Yin *et al.* 2006 | Fu *et al.* 2008 Hikosaka & Sugiyama 2015 Kim *et al.* 1992  Shin *et al.* 2007 |
| *Cucurbita maxima, C. mixta, C. moschata, C. pepo* | Pumpkin, Squash, Gourd, Marrow, Courgette | essential | #36 | Kurtar 2003  Martínez *et al.* 2014 Nogueira *et al.* 2011  Robinson & Reiners 1999 |  | Martínez *et al.* 2013 Yu 1999 |
| *Durio zibethinus* | Durian | great |  |  |  |  |
| *Eriobotrya japonica* | Loquat, Japanese plum, Japanese mediar | great |  |  | Tao et al. 2015 | **Mesejo *et al.* 2010**  Ding 1988 |
| *Fagopyrum esculentum* | Buckwheat | great |  |  |  |  |
| *Feijoa sellowania* | Feijoa | great |  |  |  |  |
| *Ficus carica* | Fig | modest |  |  |  | Blondeau and Crane 1949  Crane 1964  Crane et al. 1948 |
| *Fragaria* spp. | Strawberry | modest |  |  | **Mezzetti *et al.* 2004** | Mudge et al. 1981 |
| *Lagenaria siceraria* | Bottle gourd | Not in Klein et al. 2007 |  |  |  | **Yu 1999** |
| *Luffa acutangula* | Luffa | Not in Klein et al. 2007 |  |  |  | **Bisaria 1977** |
| *Malus domestica*  *parthenocarpy listed as one of breeding mechanisms | Apple | great | #19 | **Watanabe *et al.* 2008** | Yao *et al.* 2001 | **Watanabe *et al.* 2008**  Bangerth 1994  Bangerth et al. 1994  Bukovac 1963  Goldwin et al  Goldwin et al 1989  Greene 1980  Williams 1980  Luckwill 1960 |
| *Mangifera indica* | Mango | great | #30 |  | **Ogata *et al.* 2010** | Ogata 2009  Perez-Barraza et al. 2015 |
| *Manilkara zapota* | Sapodilla | essential |  |  |  |  |
| *Passiflora edulis* | Passionfruit | essential |  |  |  |  |
| *Persea americana* | Avocado | great |  |  |  |  |
| *Prunus armeniaca* | Apricot | great |  |  |  | Crane et al. 1960 |
| *Prunus avium* | Sweet cherry | great |  |  |  | Crane et al. 1960 |
| *Prunus cerasus* | Sour cherry | great |  |  |  | Crane et al. 1960 |
| *Prunus domestica, P. spinosa* | Plum, Greengage, Mirabelle, Sloe | great | #48 |  |  | Crane et al. 1960  Hartmann 1984  Jackson 1968 |
| *Prunus persica* | Peach, Nectarine | great | #42 |  |  | Crane et al. 1960 |
| *Psidium guajava* | Guava, Guayaba | modest |  |  |  |  |
| *Punica granatum* | Pomegranate | modest |  |  |  |  |
| *Pyrus communis* | Pear | great | #37 | Nishitani *et al.* 2012 |  | **Lafer 2008**  **Niu *et al.* 2015**  Yarushnykov & Blanke 2005  Zhang *et al.* 2008  Luckwill 1960  Yamada et al. 1991 |
| *Ribes nigrum, R. rubrum,* | Black currant, Red currant | modest |  |  |  |  |
| *Rosa spp.* | Rose hips, Dogroses | great |  |  |  |  |
| *Rubus ideaus, R. fruiticosus, R. chamaemorus, R. flagellaris, R. trivalis* | Raspberry, Blackberry, Cloudberry, Northern Drewberry, Southern Drewberry | great |  |  | **Mezzetti *et al.* 2004** | **Junttila *et al.* 2002** |
| *Solanum lycopersicum*  *parthenocarpy listed as one of breeding mechanisms | Tomato | little | #12 | **Baggett *et al.* 1997**  Charbeonboonsit *et al.* 1985  Costa *et al.* 1992  Dutta *et al.* 2013  **Fos *et al.* 2003**  Gorguet *et al.* 2005  **Habashy *et al.* 2004**  Mazzucato *et al.* 1998  Mohamed 1998  Philouze et al. 1986  Sugahara et al. 2002  Tang et al. 2015 | **Barg & Salts 2000**  Carmi *et al.* 2003  Carrera *et al.* 2012  **Ficcadenti *et al.* 1999**  **García-Hurtado *et al.* 2012**  Goetz *et al.* 2007  Ingrosso *et al.* 2011  Marti *et al.* 2007  **Medina *et al.* 2013**  **Molesini *et al.* 2009**  Pandolfini *et al.* 2002  **Rotino *et al.* 2005**  Schijlen *et al.* 2007  **Shabtai *et al.* 2007** | Fos *et al.* 2003  **Gemici *et al.* 2006**  **Goetz *et al.* 2007**  Gustafson 1936  **Karapanos *et al.* 2013**  **Nandwani *et al.* 2014**  **Ramin 2003**  Rounis *et al.* 2015  **Serrani *et al.* 2008**  Aguero et al. 2007  Alabadi et al. 1996  Alabadi et al. 1998  Ampomah-Dwamena et al. 2002  Bunger-Kibler et al. 1982  Costa et al 1985  El-Habbasha et al.1999  Gorecka et al. 1987  Gustafson 1960  Mapelli et al. 1987  Mariotti et al. 2011  Matsuo et al, 2012  Mazzucato et al. 1999  Mignolli et al. 2012  Shinozaki et al. 2015  Sjut 1982  Sjut 1984  varga et al 1986  Gelmesa et al. 2013 |
| *Solanum melongena* | Aubergine | modest | #27 | Kikuchi *et al.* 2008  Mori *et al.* 2013  **Saito *et al.* 2009**  Boyaci 2009  Takeshi et al. 2010 | **Acciarri *et al.* 2002**  **Donzella *et al.* 2000**  **Rotino *et al.* 1997** | Boyaci *et al.* 2011  Gustafson 1936  Sarma et al. 1997  Sidhu 2007 |
| *Solanum muricatum* | Pepino dulce, Sweet cucumber | Not in Klein et al. 2007 |  | Nuez et al. 1998  Prohens et al. 2002  Prophens and Nuez 2000  Rodriguez-Burruezo et al 2011 |  | **Ercan & Akilli 1996**  Maroto *et al.* 1997 |
| *Solanum quitoense* | Naranjillo | great |  |  |  |  |
| *Sorbus aucuparia* | Rowanberry | essential |  |  |  |  |
| *Vaccinium corymbosum, V. angustifolium, V. ashei, V. myrtillus* | Highbrush blueberry, Lowbrush blueberry, Rabbiteye blueberry, Bilberry | great |  |  |  | Junttila et al. 2002 |
| *Vaccinium macrocarpon, V. oxycoccus* | American cranberry, European cranberry | great |  |  |  | Devlin & Demoranville 1967 |

## **References from search**

Acciarri, N., Restaino, F., Vitelli, G., Perrone, D., Zottini, M., Pandolfini, T., *et al.* (2002). Genetically modified parthenocarpic eggplants: improved fruit productivity under both greenhouse and open field cultivation. *BMC Biotechnol.*, 2, 1–7.

Baggett, J.., Kean, D. & Mansour, N.S. (1997). “Siletz” Parthenocarpic Tomato. *HortScience*, 32, 1299–1300.

Balakbir, A., Ruiz, J. & Romero, L. (1998). Yield and fruit quality of pepper (*Capsicum annum* L.) in response to bioregulators. *HortScience*, 33, 85–87.

Barg, R. & Salts, Y. (2000). Method for the induction of genetic parthenocarpy in plants.

Bisaria, A. (1977). Effect of a morphactin on growth, sex expression, fruit-set, and yield in *Luffa acutangula. Can. J. Bot.*, 55, 752–756.

Boyaci, H.F., Oguz, A., Yazici, K.M. & Eren, A. (2011). The efficacy of endogenous gibberellic acid for parthenocarpy in eggplant (*Solanum melongena* L .). *J. Biotechnol.*, 10, 6522–6528.

Carmi, N., Salts, Y., Dedicova, B., Shabtai, S. & Barg, R. (2003). Induction of parthenocarpy in tomato via specific expression of the rolB gene in the ovary. *Planta*, 217, 726–735.

Carrera, E., Ruiz-Rivero, O., Peres, L.E.P., Atares, A. & Garcia-Martinez, J.L. (2012). Characterization of the procera tomato mutant shows novel functions of the SlDELLA protein in the control of flower morphology, cell division and expansion, and the auxin-signaling pathway during fruit-set and development. *Plant Physiol.*, 160, 1581–1596.

Carrizo, C. (2011). Fruit characteristics, seed production and pollen tube growth in the wild chilli pepper *Capsicum flexuosum*. *Flora Morphol. Distrib. Funct. Ecol. Plants*, 206, 334–340.

Charbeonboonsit, S., Splittstoesser, W. & George, W. (1985). The effects of pollination methods and auxin applications upon parthenocarpic fruit set and development in tomato. *Sci. Hortic. (Amsterdam).*, 27, 1–8.

Costa, J., Catala, S., Botella, F., Nuez, F. & Cuartero, J. (1992). Freda - a new tomato parthenocarpic hybrid. *Hortscience*, 27, 185–186.

Devlin, R.M. & Demoranville, I.E. (1967). Influence of gibberellic acid and gibrel on frnit set and yield in *Vaccinium macro- carpon* cv. Early Black. *Physiol. Plant.*, 20, 587–592.

Donzella, G., Spena, A. & Rotino, G.L. (2000). Transgenic parthenocarpic eggplants: Superior germplasm for increased winter production. *Mol. Breed.*, 6, 79–86.

Dutta, a. K., Akhtar, S., Karak, C. & Hazra, P. (2013). Gene actions for fruit yield and quality characters of tomato through generation mean analysis. *Indian J. Hortic.*, 70, 230–237.

Ercan, N. & Akilli, M. (1996). Reasons for parthenocarpy and the effects of various hormone treatments on fruit set in pepino (*Solanum muricatum* Ait.). *Sci. Hortic. (Amsterdam).*, 66, 141–147.

Ficcadenti, N., Sestili, S., Pandolfini, T., Cirillo, C., Leonardo, G. & Spena, A. (1999). Genetic engineering of parthenocarpic fruit development in tomato. *Mol. Breed.*, 5, 463–470.

Fos, M., Proaño, K., Alabadí, D., Nuez, F., Carbonell, J. & García-Martínez, J.L. (2003). Polyamine metabolism is altered in unpollinated parthenocarpic pat-2 tomato ovaries. *Plant Physiol.*, 131, 359–366.

Fu, F.Q., Mao, W.H., Shi, K., Zhou, Y.H., Asami, T. & Yu, J.Q. (2008). A role of brassinosteroids in early fruit development in cucumber. *J. Exp. Bot.*, 59, 2299–2308.

García-Hurtado, N., Carrera, E., Ruiz-Rivero, O., López-Gresa, M.P., Hedden, P., Gong, F., *et al.* (2012). The characterization of transgenic tomato overexpressing gibberellin 20 oxidase reveals induction if parthenocarpic fruit growth, higher yield, and alteration of the gibberellin biosynthetic pathway. *J. Exp. Bot.*, 63, 695–709.

García-Martínez, J. & García-Papí, M. (1979). The Influence of gibberellic acid, 2,4-D dichlorophenoxyacetic acid and 6-benzylaminopurine on fruit-set of clementine mandarin. *Sci. Hortic. (Amsterdam).*, 10, 285–293.

Gemici, M., Türkyılmaz, B. & Tan, K. (2006). Effects of 2,4-D and 4-CPA on yield and quality of the tomato, *Lycopersicon esculentum* Miller. *J. Food Sci.*, 29, 24–32.

Goetz, M., Hooper, L.C., Johnson, S.D., Rodrigues, J.C.M., Vivian-Smith, A. & Koltunow, a. M. (2007). Expression of aberrant forms of auxin response factor 8 stimulates parthenocarpy in arabidopsis and tomato. *Plant Physiol.*, 145, 351–366.

Gorguet, B., Van Heusden, a. W. & Lindhout, P. (2005). Parthenocarpic fruit development in tomato. *Plant Biol.*, 7, 131–139.

Guardiola, J.L., Barrés, M.T., Albert, C. & García-Luis, A. (1993). Effects of exogenous growth regulators on fruit development in citrus unshiu. *Ann. Bot.*

Gustafson, F.G. (1936). Inducement of fruit development by growth-promoting chemicals. *Proc. Natl. Acad. Sci. U. S. A.*, 22, 628–636.

Habashy, a. a., Testa, G., Mosconi, P., Caccia, R., Mazzucato, A., Santange-Lo, E., *et al.* (2004). Parthenocarpy restores fruitfulness in sterile triploid (3x) tomatoes artificially obtained by crossing 4x x 2x somaclones. *J. Hortic. Sci. Biotechnol.*, 79, 322–328.

Hayata, Y., Niimi, Y., Inoue, K. & Kondo, S. (2000). CPPU and BA, with and without pollination, affect set, growth, and quality of muskmelon fruit. *HortScience*, 35, 868–870.

Hayata, Y., Niimi, Y. & Iwasaki, N. (1995). Synthetic cytokinin-1-(2=chloro=4=pyridyl)-3-phenylurea (CPPU)-promotes fruit set and induces parthenocarpy in watermelon. *J. Am. Soc. Hortic. Sci.*, 120, 997–1000.

Heuvelink, E. & Korner, O. (2001). Parthenocarpic fruit growth reduces yield fluctuation and blossom-end rot in sweet pepper. *Ann. Bot.*, 88, 69–74.

Hikosaka, S. & Sugiyama, N. (2015). Effects of exogenous plant growth regulators on yield, fruit growth, and concentration of endogenous hormones in gynoecious parthenocarpic cucumber (*Cucumis sativus* L.). *Hortic. J.*, 84, 342–349.

Honda, I., Matsunaga, H., Kikuchi, K., Matsuo, S. & Fukuda, M. (2012). Identification of pepper (*Capsicum annuum* L.) accessions with large or small fruit that have a high degree of parthenocarpy. *Sci. Hortic. (Amsterdam).*, 135, 68–70.

Huitrón, M.V., Diaz, M., Diánez, F., Camacho, F. & Valverde, A. (2007). Effect of 2,4-D and CPPU on triploid watermelon production and quality. *HortScience*, 42, 559–564.

Ingrosso, I., Bonsegna, S., De Domenico, S., Laddomada, B., Blando, F., Santino, A., *et al.* (2011). Over-expression of a grape stilbene synthase gene in tomato induces parthenocarpy and causes abnormal pollen development. *Plant Physiol. Biochem.*, 49, 1092–9.

Iwahori, S., Tominaga, S. & Yamasaki, T. (1988). Stimulation of fruit growth of kiwifruit, *Actinidia chinensis* Planch., by N-(2-chloro-4-pyridyl)-N’-phenylurea, a diphenylurea-derivative cytokinin, 35, 109–115.

Junttila, O., Martinussen, I., Ernstsen, A., Nilsen, G. & Bhuvaneswari, T. V. (2002). Parthenocarpic fruit development in cloudberry (*Rubus chamaemorus* L.) is induced by 3B-hydroxylated gibberellins. *J. Hortic. Sci. Biotechnol.*, 77, 9–12.

Karapanos, I.C.., Alexopoulos, A.A.., Akoumianakis, K.A.., Grigoriou, F.., Miliordos, D.., Rigakis, K., *et al.* (2013). Application of β-naphthoxyacetic acid ( β-NOA) improves fruit yield and marketable quality in out- of-season cherry tomatoes (*Solanum lycopersicum* L. var. cerasiforme) cultivated in unheated greenhous. *J. Hortic. Sci. Biotechnol. ISSN*, 88, 165–172.

Kikuchi, K., Honda, I., Matsuo, S., Fukuda, M. & Saito, T. (2008). Stability of fruit set of newly selected parthenocarpic eggplant lines. *Sci. Hortic. (Amsterdam).*, 115, 111–116.

Kim, I.S., Okubo, H. & Fujieda, K. (1992). Endogenous levels of IAA in relation to parthenocarpy in cucumber (*Cucumis sativus* L.). *Sci. Hortic. (Amsterdam).*, 52, 1–8.

Klein, A.-M., Vaissière, B.E., Cane, J.H., Steffan-Dewenter, I., Cunningham, S. A, Kremen, C., *et al.* (2007). Importance of pollinators in changing landscapes for world crops. *Proc. R. Soc. B*, 274, 303–313.

Kurtar, E.. (2003). An investigation of parthenocarpy in some summer squash (*Cucurbita pepo* L.) Cultivars. *Pakistan J. Agron.*, 2, 209–213.

Kushnereva, V. (2008). Breeding of cucumber (*Cucumis sativus*) for resistance to multiple diseases and other traits. In: *Proceedings of the IXth EUCARPIA meeting on genetics and breeding of Cucurbitaceae*. pp. 429–432.

Kwon, S.W., Jaskani, M.J. & Ko, B.R. (2006). Evaluation of soft x-ray irradiated pollen and CPPU for diploid seedless watermelon production. *Acta Hortic.*, 289–294.

Lafer, G. (2008). Effects of different bioregulator applications on fruit set, yield and fruit quality of “Williams” pears. *Acta Hortic.*, 183–187.

Li, J., Wu, Z., Cui, L., Zhang, T., Guo, Q., Xu, J., *et al.* (2014). Transcriptome comparison of global distinctive features between pollination and parthenocarpic fruit set reveals transcriptional phytohormone cross-talk in cucumber (*Cucumis sativus* L.). *Plant Cell Physiol.*, 55, 1325–1342.

Lora, J., Hormaza, J.I., Herrero, M. & Gasser, C.S. (2011). Seedless fruits and the disruption of a conserved genetic pathway in angiosperm ovule development. *Proc. Natl. Acad. Sci. U. S. A.*, 108, 5461–5465.

Maroto, J. V., Miguel, A., Lopez-Galarza, S., San Bautista, A., Pascual, B., Alagarda, J., *et al.* (2005). Parthenocarpic fruit set in triploid watermelon induced by CPPU and 2,4-D applications. *Plant Growth Regul.*, 45, 209–213.

Maroto, J.V., Lopez-Galarza, S., Pascual, B., Bono, M.S., San Bautista, A. & Alagarda, J. (1997). Procarpil enhances earliness and parthenocarpy of pepino (*Solanum muricatum* Ait.). *HortScience*, 32, 133.

Marti, C., Orzaez, D., Ellul, P., Moreno, V., Carbonell, J. & Granell, A. (2007). Silencing of DELLA induces facultative parthenocarpy in tomato fruits. *Plant J.*, 52, 865–876.

Martínez, C., Manzano, S., Megías, Z., Garrido, D., Picó, B. & Jamilena, M. (2013). Involvement of ethylene biosynthesis and signalling in fruit set and early fruit development in zucchini squash (*Cucurbita pepo* L.). *BMC Plant Biol.*, 13, 1–14.

Martínez, C., Manzano, S., Megías, Z., Garrido, D., Picó, B. & Jamilena, M. (2014). Sources of parthenocarpy for zucchini breeding: relationship with ethylene production and sensitivity. *Euphytica*, 200, 349–362.

Mazzucato, a, Taddei, a R. & Soressi, G.P. (1998). The parthenocarpic fruit (pat) mutant of tomato (*Lycopersicon esculentum* Mill.) sets seedless fruits and has aberrant anther and ovule development. *Development*, 125, 107–114.

Medina, M., Roque, E., Pineda, B., Cañas, L., Rodriguez-Concepción, M., Beltrán, J.P., *et al.* (2013). Early anther ablation triggers parthenocarpic fruit development in tomato. *Plant Biotechnol. J.*, 11, 770–9.

Mesejo, C., Reig, C., Martínez-Fuentes, A. & Agustí, M. (2010). Parthenocarpic fruit production in loquat (*Eriobotrya japonica* Lindl.) by using gibberellic acid. *Sci. Hortic. (Amsterdam).*, 126, 37–41.

Mesejo, C., Yuste, R., Martinez-Fuentes, A., Reig, C., Iglesias, D.J., Primo-Millo, E., *et al.* (2013). Self-pollination and parthenocarpic ability in developing ovaries of self-incompatible clementine mandarins (*Citrus clementina*). *Physiol. Plant.*, 148, 87–96.

Mezzetti, B., Landi, L., Pandolfini, T. & Spena, A. (2004). The defH9-iaaM auxin-synthesizing gene increases plant fecundity and fruit production in strawberry and raspberry. *BMC Biotechnol.*, 4, 4.

Mohamed, M.F. (1998). Characteristics and inheritance of natural facultative-parthenocarpic fruit-set in “Nadja” tomato under low temperature conditions. *Euphytica*, 103, 211–217.

Molesini, B., Pandolfini, T., Rotino, G.L., Dani, V. & Spena, A. (2009). Aucsia gene silencing causes parthenocarpic fruit development in tomato. *Plant Physiol.*, 149, 534–548.

Mori, T., Umeda, T., Honda, T., Zushi, K., Wajima, T. & Matsuzoe, N. (2013). Varietal differences in the chlorogenic acid, anthocyanin, soluble sugar, organic acid, and amino acid concentrations of eggplant fruit. *J. Hortic. Sci. Biotechnol.*, 88, 657–663.

Nandwani, D., Dennery, S. & Balkaran, S. (2014). Effect of 4-CPA on fruit Set and yield of beefsteak tomato (*Solanum lycopersicum* L .) on the Island of St. Croix. *Acta Hortic.*, 255–260.

Nishitani, C., Yamaguchi-Nakamura, A., Hosaka, F., Terakami, S., Shimizu, T., Yano, K., *et al.* (2012). Parthenocarpic genetic resources and gene expression related to parthenocarpy among four species in pear (*Pyrus* spp.). *Sci. Hortic. (Amsterdam).*, 136, 101–109.

Niu, Q., Wang, T. & Li, J. (2015). Effects of exogenous application of GA4+7 and N-(2-chloro-4-pyridyl)-N’-phenylurea on induced parthenocarpy and fruit quality in *Pyrus pyrifolia* “Cuiguan.” *Plant Growth Regul.*, 251–258.

Nogueira, D.W., Maluf, W.R., Dos Reis Figueira, A., Maciel, G.M., Gomes, L.A.A. & Benavente, C.A.T. (2011). Combining ability of summer-squash lines with different degrees of parthenocarpy and PRSV-W resistance. *Genet. Mol. Biol.*, 34, 616–23.

Ogata, T., Tamura, H., Hamada, K. & Hasegawa, K. (2010). Effect of gibberelin on setting and growth of non-pollinated parthenocarpic fruit in mango, 597–604.

Pandolfini, T., Rotino, G.L., Camerini, S., Defez, R. & Spena, A. (2002). Optimisation of transgene action at the post-transcriptional level: high quality parthenocarpic fruits in industrial tomatoes. *BMC Biotechnol.*, 2.

Ramin, a a. (2003). Effects of auxin application on fruit formation in tomato growing under stress temperatures in the field. *J. Hortic. Sci. Biotechnol.*, 78, 706–710.

Rimberia, F.K., Adaniya, S., Ishimine, Y. & Etoh, T. (2007). Morphology of papaya plants derived via anther culture. *Sci. Hortic. (Amsterdam).*, 111, 213–219.

Risser, G. (1976). Induction of parthenocarpy in *Cucumis melo* by “PH 30.13.” *Sci. Hortic. (Amsterdam).*, 5, 73–76.

Robinson, R.W. & Reiners, S. (1999). Parthenocarpy in summer squash. *HortScience*, 34, 715–717.

Rotino, G.L., Acciarri, N., Sabatini, E., Mennella, G., Lo Scalzo, R., Maestrelli, A., *et al.* (2005). Open field trial of genetically modified parthenocarpic tomato: seedlessness and fruit quality. *BMC Biotechnol.*, 5.

Rotino, G.L., Perri, E., Zottini, M., Sommer, H. & Spena, A. (1997). Genetic engineering of parthenocarpic plants. *Nat. Biotechnol.*, 15, 1398–1401.

Rounis, V., Skarmoutsos, K., Tsaniklidis, G., Nikoloudakis, N., Delis, C., Karapanos, I., *et al.* (2015). Seeded and parthenocarpic cherry tomato fruits exhibit similar sucrose, glucose, and fructose levels, despite dissimilarities in UGPase and SPS gene expression and enzyme activity. *J. Plant Growth Regul.*, 34, 47–56.

Saito, T., Yoshida, T., Monma, S., Matsunaga, H., Sato, T., Saito, A., *et al.* (2009). Development of the parthenocarpic eggplant cultivar “Anominori.” *Japan Agric. Res. Q.*, 43, 123–127.

Schijlen, E.G.W.M., de Vos, C.H.R., Martens, S., Jonker, H.H., Rosin, F.M., Molthoff, J.W., *et al.* (2007). RNA interference silencing of chalcone synthase, the first step in the flavonoid biosynthesis pathway, leads to parthenocarpic tomato fruits. *Plant Physiol.*, 144, 1520–1530.

Sedgley, M., Newbury, H.J. & Possingham, J. V. (1977). Early fruit development in the watermelon: Anatomical comparison of pollinated, auxin-induced parthenocarpic and unpollinated fruits. *Ann. Bot.*, 1345–1355.

Serrani, J.C., Ruiz-Rivero, O., Fos, M. & García-Martínez, J.L. (2008). Auxin-induced fruit-set in tomato is mediated in part by gibberellins. *Plant J.*, 56, 922–934.

Shabtai, S., Salts, Y., Kaluzky, G. & Barg, R. (2007). Improved yielding and reduced puffiness under extreme temperatures induced by fruit-specific expression of rolB in processing tomatoes. *Theor. Appl. Genet.*, 114, 1203–1209.

Shaw, N., Cantliffe, D. & Stofella, P. (2007). A new crop for North American greenhouse growers: Beit alpha cucumber - progress of production technology through university research trials. *Acta Hortic.*, 731, 251–258.

Shin, Y.S., Park, S.D. & Kim, J.H. (2007). Influence of pollination methods on fruit development and sugar contents of oriental melon (*Cucumis melo* L. cv. Sagyejeol-Ggul). *Sci. Hortic. (Amsterdam).*, 112, 388–392.

Sun, Z., Lower, R.L. & Staub, J.E. (2006). Variance component analysis of parthenocarpy in elite U.S. processing type cucumber (*Cucumis sativus* L.) lines. *Euphytica*, 148, 331–339.

Thanopoulos, C., Bouranis, D. & Passam, H.C. (2013). Comparative development, maturation and ripening of seedless and seed-containing bell pepper fruits. *Sci. Hortic. (Amsterdam).*, 164, 573–577.

Tiwari, a., Dassen, H. & Heuvelink, E. (2007). Selection of sweet pepper (*Capsicum annuum* L.) genotypes for parthenocarpic fruit growth. *Acta Hortic.*, 2007, 135–140.

Tiwari, A., Offringa, R. & Heuvelink, E. (2012). Auxin-induced fruit set in *Capsicum annuum* L. requires downstream gibberellin biosynthesis. *J. Plant Growth Regul.*, 31, 570–578.

Watanabe, M., Segawa, H., Murakami, M., Sagawa, S. & Komori, S. (2008). Effects of plant growth regulators on fruit set and fruit shape of parthenocarpic apple fruits. *J. Japanese Soc. Hortic. Sci.*, 77, 350–357.

Wien, H.C. & Zhang, Y. (1991). Prevention of flower abscission in bell pepper. *J. Am. Soc. Hortic. Sci.*, 16, 516–519.

Yan, L.Y., Lou, L.N., Li, X.L., Feng, Z.H., Lou, Q.F. & Chen, J.F. (2012). Inheritance of parthenocarpy in cucumber under the same background. *Acta Hortic.*, 935, 55–59.

Yao, J., Dong, Y. & Morris, B. a. (2001). Parthenocarpic apple fruit production conferred by transposon insertion mutations in a MADS-box transcription factor. *Proc. Natl. Acad. Sci. U. S. A.*, 98, 1306–1311.

Yarushnykov, V. V. & Blanke, M.M. (2005). Alleviation of frost damage to pear flowers by application of gibberellin. *Plant Growth Regul.*, 45, 21–27.

Yin, Z., Malinowski, R., Ziółkowska, A., Sommer, H., Plcader, W. & Malepszy, S. (2006). The DefH9-iaaM-containing construct efficiently induces parthenocarpy in cucumber. *Cell. Mol. Biol. Lett.*, 11, 279–290.

Yu, J. (1999). Parthenocarpy induced by N-(2-chloro-4-pyridyl)-N’-phenylurea (CPPU) prevents flower abortion in Chinese white-flowered gourd (*Lagenaria leucantha*). *Environ. Exp. Bot.*, 42, 121–128.

Zhang, C., Lee, U. & Tanabe, K. (2008). Hormonal regulation of fruit set, parthenogenesis induction and fruit expansion in Japanese pear. *Plant Growth Regul.*, 55, 231–240.
