# Supplementary material for: Re‐evaluating strategies for pollinator‐dependent crops: How useful is parthenocarpy?
Source: J Appl Ecol. 2016 Nov 11;54(4):1171–9. doi: 10.1111/1365-2664.12813 (PMC5516152; doi:10.1111/1365-2664.12813)
Supplement: Supplementary file 3 — Table S2. Final dataset used in the meta‐analysis, alphabetically ordered by genus. [file JPE-54-1171-s003.docx]

## **Table S2** Final dataset used in the meta-analysis, alphabetically ordered by genus.

| **Species** | **Method to induce Parthenocarpy** | **Test environment** | **Measure** | **Effect Size** | **Variance** |
| --- | --- | --- | --- | --- | --- |
| *Actinidia chinensis* | HA | NP conditions | Sugars | -0.082 | 0.021109 |
| *Actinidia chinensis* | HA | NP conditions | Yield | 1.996201 | 0.245295 |
| *Capsicum annuum* | SB | OP conditions | Yield | 1.836082 | 0.028712 |
| *Capsicum annuum* | HA | NP conditions | Yield | 1.33715 | 0.005758 |
| *Capsicum annuum* | HA | HP conditions | Yield | -0.34255 | 0.012835 |
| *Carica papaya* | SB | OP conditions | Yield | 1.136643 | 0.003864 |
| *Citrullus lanatus* | HA | HP conditions | Sugars | -0.20544 | 0.006123 |
| *Citrullus lanatus* | HA | HP conditions | Yield | 0.155029 | 0.006113 |
| *Citrullus lanatus* | HA | OP conditions | Sugars | 0.051341 | 0.021092 |
| *Citrullus lanatus* | HA | OP conditions | Yield | -0.92558 | 0.007337 |
| *Cucumis melo* | HA | NP conditions | Sugars | 13.91252 | 0.159346 |
| *Cucumis melo* | HA | NP conditions | Yield | 10.83453 | 0.099127 |
| *Cucumis melo* | HA | OP conditions | Sugars | 0.460481 | 0.006492 |
| *Cucumis melo* | HA | OP conditions | Yield | 0.275551 | 0.006385 |
| *Eriobotrya japonica* | HA | OP conditions | Sugars | 0.086106 | 0.001266 |
| *Eriobotrya japonica* | HA | OP conditions | Yield | -2.20575 | 0.002034 |
| *Fragaria ananassa* | GM | OP conditions | Sugars | 0.057971 | 0.002531 |
| *Fragaria ananassa* | GM | OP conditions | Yield | 0.91286 | 0.001397 |
| *Fragaria vesca* | GM | OP conditions | Sugars | 0.164231 | 0.006346 |
| *Fragaria vesca* | GM | OP conditions | Yield | 2.621295 | 0.003919 |
| *Lagenaria siceraria* | HA | NP conditions | Yield | 2.876362 | 0.003216 |
| *Luffa acutangula* | HA | OP conditions | Yield | 1.4368 | 0.007957 |
| *Malus pumila* | HA | NP conditions | Yield | 4.924251 | 0.012747 |
| *Malus pumila* | SB | NP conditions | Yield | 7.911708 | 3.269794 |
| *Mangifera indica* | HA | OP conditions | Sugars | 0.81371 | 0.006848 |
| *Mangifera indica* | HA | OP conditions | Yield | 1.687746 | 0.008576 |
| *Pyrus communis* | HA | OP conditions | Yield | 0.582146 | 0.001236 |
| *Pyrus communis* | HA | OP conditions | Yield | 0.393657 | 0.001201 |
| *Rubus chamaemorus* | HA | HP conditions | Yield | 0.121166 | 0.004547 |
| *Rubus idaeus* | GM | OP conditions | Sugars | 2.470727 | 0.005575 |
| *Rubus idaeus* | GM | OP conditions | Yield | 0.798029 | 0.001707 |
| *Solanum lycopersicum* | SB | OP conditions | Yield | 5.113391 | 0.003374 |
| *Solanum lycopersicum* | GM | OP conditions | Sugars | 0.440201 | 0.006922 |
| *Solanum lycopersicum* | GM | OP conditions | Yield | 0.605605 | 0.007052 |
| *Solanum lycopersicum* | GM | HP conditions | Sugars | 1.283145 | 0.005336 |
| *Solanum lycopersicum* | GM | HP conditions | Yield | 1.650157 | 0.006492 |
| *Solanum lycopersicum* | GM | NP conditions | Sugars | 15.43655 | 131.9856 |
| *Solanum lycopersicum* | GM | NP conditions | Yield | 10.62201 | 12.64204 |
| *Solanum lycopersicum* | HA | NP conditions | Yield | 3.61653 | 0.015346 |
| *Solanum lycopersicum* | SB | NP conditions | Yield | 3.204817 | 0.013882 |
| *Solanum lycopersicum* | GM | OP conditions | Sugars | 1.425102 | 0.01586 |
| *Solanum lycopersicum* | GM | OP conditions | Yield | 1.991905 | 0.009461 |
| *Solanum lycopersicum* | GM | OP conditions | Sugars | 3.43146 | 0.031267 |
| *Solanum lycopersicum* | GM | OP conditions | Yield | 1.070159 | 0.00723 |
| *Solanum lycopersicum* | HA | OP conditions | Yield | 1.336772 | 0.007737 |
| *Solanum lycopersicum* | GM | NP conditions | Yield | 2.094612 | 0.001646 |
| *Solanum lycopersicum* | SB | OP conditions | Yield | 0.484457 | 0.004744 |
| *Solanum lycopersicum* | HA | OP conditions | Yield | 3.244583 | 0.185242 |
| *Solanum lycopersicum* | GM | OP conditions | Sugars | 11.85771 | 0.234966 |
| *Solanum lycopersicum* | GM | OP conditions | Yield | 8.835926 | 0.136094 |
| *Solanum lycopersicum* | GM | HP conditions | Yield | -3.62953 | 0.014606 |
| *Solanum lycopersicum* | GM | NP conditions | Yield | 36.08369 | 1.093537 |
| *Solanum lycopersicum* | HA | OP conditions | Yield | 1.186435 | 0.038214 |
| *Solanum lycopersicum* | GM | NP conditions | Yield | 2.020992 | 0.009014 |
| *Solanum lycopersicum* | HA | OP conditions | Yield | 4.556189 | 0.631613 |
| *Solanum lycopersicum* | GM | OP conditions | Sugars | 0.210216 | 0.000111 |
| *Solanum lycopersicum* | GM | OP conditions | Yield | 0.143855 | 0.00317 |
| *Solanum lycopersicum* | HA | NP conditions | Yield | 28.83752 | 0.829705 |
| *Solanum lycopersicum* | GM | NP conditions | Sugars | -1.42147 | 0.001866 |
| *Solanum lycopersicum* | GM | NP conditions | Yield | 1.739234 | 0.006145 |
| *Solanum melongena* | GM | OP conditions | Yield | 1.444628 | 0.070885 |
| *Solanum melongena* | SB | OP conditions | Yield | 1.761513 | 0.01627 |
| *Solanum melongena* | GM | NP conditions | Yield | 3.679541 | 1.106564 |
| *Solanum melongena* | HA | NP conditions | Yield | 2.399622 | 0.121138 |
| *Solanum melongena* | SB | NP conditions | Yield | -1.19352 | 0.016926 |
| *Solanum melongena* | GM | NP conditions | Yield | 4.184441 | 1.306233 |
| *Solanum melongena* | SB | OP conditions | Yield | 2.084884 | 0.007034 |
| *Solanum muricatum* | HA | OP conditions | Yield | 1.662862 | 0.037947 |
| *Solanum muricatum* | HA | OP conditions | Yield | 0.237487 | 0.004253 |
